# Prevention of Disease

**Published:** 1882-05

**Authors:** 


### PREVENTION OF DISEASE.

"An ounce of prevention is worth a pound of cure." We should have a very healthy world if everybody heeded this old saying. The very worst diseases of all—yellow fever, cholera, scarlet fever, diphtheria, typhoid and typus fevers, and most diarrhœal complaints—would soon be eradicated. And this could be done simply by everybody's keeping everything in and around his premises clean. But this cleanliness demands more than washing and scrubbing. It requires that all waste, both of the premises and the person, should be at once removed to distant places of deposit by the most approved methods of conveyance; a work possible, except in rural districts, only by the united effort of the community. Even consumption, which now kills every eighth person, is largely a preventable disease. Although it is peculiarly subject to the law of heredity, yet intelligent and persistent care will not only arrest the consumptive tendency, but in time eradicate it. The prime thing, however, is to prevent the original taint; and there is no doubt that this is possible far beyond popular belief. It is now known that the disease can be contracted by breathing the unventilated air of the patient's room, or by drinking the milk or eating the flesh of tuberculous cows; that whatever lowers a person's vitality supplies an essential condition of its development; and that damp localities and changeable climates are either its chief producing or exciting cause. In relation to all these points, however, we need to have our knowledge greatly increased; and State boards of health should be furnished with means for combining their efforts to secure all essential facts and statistics. and for securing, to the fullest extent, every essential condition of public hygiene.

